# COVID-19 and Chronic Disease: The Impact Now and in the Future

**DOI:** 10.5888/pcd18.210086

**Published:** 2021-06-17

**Authors:** Karen A. Hacker, Peter A. Briss, Lisa Richardson, Janet Wright, Ruth Petersen

**Affiliations:** 1 National Center for Chronic Disease Prevention and Health Promotion, Centers for Disease Control and Prevention, Atlanta, Georgia

## The Problem of COVID-19 and Chronic Disease

Chronic diseases represent 7 of the top 10 causes of death in the United States (1). Six in 10 Americans live with at least 1 chronic condition, such as heart disease, stroke, cancer, or diabetes (2). Chronic diseases are also the leading causes of disability in the US and the leading drivers of the nation’s $3.8 trillion annual health care costs (2,3).

The COVID-19 pandemic has resulted in enormous personal and societal losses, with more than half a million lives lost (4). COVID-19 is a disease caused by severe acute respiratory syndrome coronavirus 2 (SARS-CoV-2) that can result in respiratory distress. In addition to the physical toll, the emotional impact has yet to be fully understood. For those with chronic disease, the impact has been particularly profound (5,6). Heart disease, diabetes, cancer, chronic obstructive pulmonary disease, chronic kidney disease, and obesity are all conditions that increase the risk for severe illness from COVID-19 (7). Other factors, including smoking and pregnancy, also increase the risk (7). Finally, in addition to COVID-19–related deaths since February 1, 2020, an increase in deaths has been observed among people with dementia, circulatory diseases, and diabetes among other causes (8). This increase could reflect undercounting COVID-19 deaths or indirect effects of the virus, such as underutilization of, or stresses on, the health care system (8).

Some populations, including those with low socioeconomic status and those of certain racial and ethnic groups, including African American, Hispanic, and Native American, have a disproportionate burden of chronic disease, SARS-CoV-2 infection, and COVID-19 diagnosis, hospitalization, and mortality (9). These populations are at higher risk because of exposure to suboptimal social determinants of health (SDoH). SDoH are factors that influence health where people live, work, and play, and can create obstacles that contribute to inequities. Education, type of employment, poor or no access to health care, lack of safe and affordable housing, lack of access to healthy food, structural racism, and other conditions all affect a wide range of health outcomes (10–12). The COVID-19 pandemic has exacerbated existing health inequities and laid bare underlying root causes.

The COVID-19 pandemic has had direct and indirect effects on people with chronic disease. In addition to morbidity and mortality, high rates of community spread and various mitigation efforts, including stay-at-home recommendations, have disrupted lives and created social and economic hardships (13). This pandemic has also raised concerns about safely accessing health care (14) and has reduced the ability to prevent or control chronic disease. This essay discusses the impact that these challenges have or could have on people with chronic disease now and in the future. Exploring the impact of COVID-19 should help the public health and health care communities effectively improve health outcomes.

## Challenges

The challenges we face as public health professionals are divided into 3 categories. The first category involves the current effects of COVID-19 on those with, or at risk for, chronic diseases and those at higher risk for severe COVID-19 illness. Inherent in this category is the need for balance between protecting people with chronic diseases from COVID-19 while assuring they can engage in disease prevention, manage their conditions effectively, and safely receive needed health care.

The second category is the postpandemic impact of COVID-19 on the prevention, identification, and management of chronic disease. COVID-19 has resulted in decreases of many types of health care utilization (15), ranging from preventive care to chronic disease management and even emergency care (16). As of June 2020, 4 in 10 adults surveyed reported delaying or avoiding routine or emergent medical care because of the pandemic (14). Cancer screenings, for example, dropped during the pandemic (17). Decreases in screening have resulted in the diagnoses of fewer cancers and precancers (18), and modeling studies have estimated that delayed screening and treatment for breast and colorectal cancer could result in almost 10,000 preventable deaths in the United States (19). We have lost ground in prevention across the chronic disease spectrum and in other areas, including pediatric immunization (20), mental health (21,22), and substance abuse (21,22).

Some challenges with health care utilization may be improving, but improvement has not been consistent across all health care visit types, providers, patients, or communities (15). Questions about the impact of the pandemic on chronic disease include:

- What diseases have been missed or allowed to worsen?
- What is the status of prevention and disease management efforts?
- Have prevention and disease management efforts been affected by concerns such as job loss, loss of insurance, lack of access to healthy food, or loss of places and opportunities to be physically active?
- How have effects of the pandemic on health care systems (staff reductions, health practice closures, disrupted services) (23) and public health organizations’ deployment of personnel away from ongoing chronic disease prevention efforts been experienced nationally?

The effects of COVID-19, whether negative or positive, on health care and public health systems will certainly affect those with chronic disease. To fully understand the consequences of the pandemic, we need to assess its overall impact on incidence, management, and outcomes of chronic disease. This is particularly salient in communities where health inequities are already rampant or communities that are remote or underserved. Will our postpandemic response be strong enough to mitigate the exacerbation of inequities that have occurred? Can public health agencies effectively build trust in science and community health care systems where trust might never have been fully established or where it has been lost?

The third category relates to the long-term COVID-19 sequelae, both as a disease entity and from a population perspective. Has COVID-19 created a new group of patients with chronic diseases, neurologic or psychiatric conditions, diabetes, or effects on the heart, lungs, kidneys, or other organs (24)? Has it worsened existing conditions or caused additional chronic disease? And, at the population level, have the incidence and prevalence of chronic diseases increased because of pandemic-related health behaviors or other challenges, such as decreased food and nutrition security?

Given the rollout of COVID-19 vaccines and the coming end of the pandemic, this is an important time to examine the impact of COVID-19. Solutions at all levels are needed to improve health outcomes and lessen health inequities among people with or at risk for chronic disease. Solutions are likely to include increasing awareness about prevention and care during and after the pandemic, building or enhancing cross-organizational and cross-sector partnerships, innovating to address identified gaps, and addressing SDoH to improve health and achieve equity. So, what can be done?

## Raise Awareness

Additional focus is required on several aspects of awareness about the impact of COVID-19. First, public health and health care practitioners need to allay people’s fears and help them safely return to health care. We need to reemphasize chronic disease prevention and care, explain how to safely access care, and convey the host of mitigation efforts made by health care systems, providers, and public health to ensure that environments are safe (eg, mask requirements, social distancing). Emphasis on safety and mitigation applies to both disease prevention (such as encouraging healthy nutrition and physical activity, screening for cancer and other conditions, and getting oral health care) and disease management (eg, educating patients about medications to control hypertension, diabetes, asthma, and other chronic conditions). Efforts must also include helping those with chronic diseases obtain access to and gain confidence in the COVID-19 vaccine. Given current community rates of COVID-19 and the need to reenter care after the height of the pandemic, information can help patients make informed choices about the need for in-person care, communication at a distance, or temporary delays in care that is more discretionary.

To garner support to help affected communities, there is a need to build awareness about how COVID-19 has disproportionately affected particular communities, including the unequal distribution of disease, morbidity, mortality, and resources, such as access to vaccines. Awareness is dependent on access to data at the granular geographic level, including information on the burden of chronic disease and the status of SDoH. Communities need data to effectively address health inequities in the aftermath of the pandemic.

## Collaborate on Solutions and Build Trust

Public health plays a significant role in addressing health behaviors (healthy eating, physical activity, avoiding tobacco and other substance use) and community solutions to address SDoH that impact prevention and control of chronic disease. Collaborations at both the individual and system levels, however, are required for success. Collaborative partners include other government and nongovernmental organizations, health care organizations, insurers, nonprofit organizations, community and faith-based groups, schools, businesses, and others. Coalitions and community groups are critical change agents. They have worked with local health departments and others to identify solutions, bring residents into discussions, and implement action. We can learn from them about how best to build trust and foster the innovation they are leading. Solutions must also include direct discussions with residents in affected communities to understand their priorities and effectively address their concerns. These relationships are particularly salient to address SDoH. These factors have been amplified as a direct consequence of COVID-19 and will require a multisector approach to problem solving.

To achieve this will require building trust in both the health care system and the public health system. The pandemic has taken a toll on an already fragile relationship between communities and public health and health care institutions where trust has been absent or insufficient. To begin to address the trust challenge will require investments in outreach, engagement, and transparency. Conversations need to be bidirectional, long-term, and conducted by people who are trusted, who are respectful, and who can identify with affected populations.

## Innovate

Creative solutions are needed to engage populations and promote resiliency among those who are disproportionately affected by COVID-19. Efforts that need to be further developed and brought to scale include the following:

- Leveraging technology to expand the reach of health care and health promotion (eg, telemedicine, virtual program delivery, wearables, mobile device applications).
- Providing more services in community settings, as is increasingly modeled in the National Diabetes Prevention Program (25).
- Using community health workers to assist in assessing current conditions and connecting to community resources.
- Further enhancing approaches to increase access to and convenience of services (eg, increasing access to home screenings, such as cancer screening) or monitoring (eg, home blood pressure monitoring) where appropriate.

Health care approaches, such as telemedicine, have expanded greatly during the pandemic and seem likely to continue expansion over time. As these and related efforts grow, practitioners will need to ensure that existing disparities are not magnified. Care is needed to ensure that those with the highest health needs can access services. For example, are technological solutions easily accessible, available in multiple languages, compatible with readily available hardware options, such as telephones rather than laptops? Are culturally appropriate resources available to help people use and value these technologies? In addition, computer availability and internet access will need to be expanded. Challenges such as unemployment, food insecurity, limited transportation, substance abuse, and social isolation will require a multisector effort uniquely adapted to local contexts. To begin, health equity–focused policy analyses and health impact assessments will help policy makers understand better how proposed SDoH-related action might either exacerbate or mitigate chronic disease inequities. These actions will help us develop a deeper understanding of what individual communities need to mobilize and build resilience for the future. We face serious public health and population health concerns that should be the focus in the near term — particularly as equitable access to COVID-19 vaccines is a consideration in every community across the nation. We clearly have an enormous amount of work to do as we enter recovery from the pandemic, but with recovery comes enormous opportunity.

## Address Long-Term COVID-19 Sequelae

A challenge related to long-term COVID-19 sequelae is that we do not know yet the extent that COVID-19 exacerbates chronic disease, causes chronic disease, or will be determined a chronic disease unto itself. Those interested in chronic disease prevention and management need to follow the research to understand better the role they will play with this emerging situation. Long-term studies and longitudinal surveillance will help clarify these issues, and there is much research to be done. The duty of the public health community is to help ensure that the most important issues from the perspectives of patients, providers, health care, and public health systems are addressed; that potential solutions are developed and tested; and that eventual solutions are delivered where they are needed most.

## How Will the National Center for Chronic Disease Prevention and Health Promotion Contribute?

As the US enters the next phase of pandemic response, the work of National Center for Chronic Disease Prevention and Health Promotion (NCCDPHP) of the Centers for Disease Control and Prevention is evolving to address health inequities and drive toward health equity with a multipronged approach. This approach includes enhanced access to data at the local level, a focus on SDoH including a shift in the Notice of Funding Opportunity process that emphasizes a health equity lens, and an expansion of partnerships and communications.

Placing data in the hands of communities is critical for local coalitions to determine their burden of chronic disease and COVID-19, their access to resources, and the best policies and practices to implement. Data will be useful for local public health, governments, and health care systems, but can also help human services, planning, and economic development organizations. An initial step is making available data from the PLACES Project (26), which provides data on 27 chronic disease measures at the census tract level, allowing communities to understand their own chronic disease burden. In addition, modules on SDoH are in development to enhance NCCDPHP data surveillance systems. This will increase the ability to overlay chronic disease data and SDoH data at the community level. The need is also a great for core SDoH measures that allow comparisons of related outcomes across communities. NCCDPHP can augment this effort by contributing to and amplifying the SDoH measures identified for Healthy People 2030 (27).

NCCDPHP is focusing on supporting and stimulating SDoH efforts by concentrating on 5 major areas: built environment, social connectedness, food and nutrition security, tobacco policies, and connections to clinical care. For example, SDoH are the foci of recent Notices of Funding Opportunities (available at https://www.grants.gov). NCCDPHP supports multisector partnerships in numerous funding announcements and launched a joint effort with the Association of State and Territorial Health Officials and the National Association of County and City Health Officials to identify best practices in multisector collaboration to address SDoH (28). Evidence will help build a standard for success to support local coalitions in their work. States and local communities are sites of innovation, and promoting lessons learned can help build broader efforts. To address urgent needs and facilitate change, NCCDPHP must link with other sectors outside of public health and health care. The work to evaluate these efforts and determine the most effective strategies to address SDoH, therefore, will be integrated fully into NCCDPHP.

An expansion of the Racial and Ethnic Approaches to Community Health (REACH) Program (29) and other programs that address health inequities will help to target resources where they are needed most. REACH and a recently released investment in community health workers (30) demonstrate NCCDPHP’s commitment to connecting with populations that are disproportionately affected by chronic disease at the local level. These efforts are aimed at addressing the ramifications of COVID-19 while also amplifying chronic disease prevention efforts. NCCDPHP also intends to enhance the use of a health equity lens, among other approaches, to determine the best use of resources and to help assess outcomes in all programmatic activities.

Finally, communication about the impact of COVID-19 on chronic disease, returning to care, and the extent of health inequities is critical to building trust. Efforts under way include a television and digital media campaign aiming to encourage those with chronic disease to return safely to care (31). In addition to expanding work with partner organizations, both external and internal to government, NCCDPHP will embrace new ways of garnering input from affected communities. Successes and failures experienced by communities during the pandemic will continue to be of the utmost importance to NCCDPHP. In addition, important insights gained from working closely with affected communities will help NCCDPHP continually refine its national chronic disease prevention and control goals and objectives. Activities related to SDoH and health equity, data, and communication will address difficult questions now and into the future. These efforts can only be successful with collaboration and partnerships across multiple sectors.

## Conclusion

The impact of SARS-CoV-2, the virus that causes COVID-19, on people with or at risk for chronic disease cannot be overstated. COVID-19 has impeded chronic disease prevention and disrupted disease management. The problems and solutions outlined here are critically important to help those committed to chronic disease prevention and intervention to identify ways forward.

NCCDPHP is adjusting, preparing, and implementing multiple strategies to address the future. Although the work will be challenging, opportunities abound. NCCDPHP is committed to working with the health care community and a variety of partners at federal, state, and local levels to help address the realities of the post-COVID era.
